# Age, race, insurance type, and digital divide index are associated with video visit completion for patients seen for oncologic care in a large hospital system during the COVID-19 pandemic

**DOI:** 10.1371/journal.pone.0277617

**Published:** 2022-11-17

**Authors:** Matthew M. Cousins, Monica Van Til, Emma Steppe, Sophia Ng, Chandy Ellimoottil, Yilun Sun, Matthew Schipper, Joseph R. Evans

**Affiliations:** 1 Department of Radiation Oncology, University of Michigan, Ann Arbor, Michigan, United States of America; 2 Department of Biostatistics, University of Michigan, Ann Arbor, Michigan, United States of America; 3 Institute for Healthcare Policy and Innovation, University of Michigan, Ann Arbor, Michigan, United States of America; 4 Department of Urology, University of Michigan, Ann Arbor, Michigan, United States of America; LSU Health Sciences Center New Orleans: Louisiana State University Health Sciences Center, UNITED STATES

## Abstract

**Introduction:**

The COVID-19 pandemic drove rapid adoption of telehealth across oncologic specialties. This revealed barriers to telehealth access and telehealth-related disparities. We explored disparities in telehealth access in patients with cancer accessing oncologic care.

**Materials/Methods:**

Data for all unique patient visits at a large academic medical center were acquired pre- and intra-pandemic (7/1/2019-12/31/2020), including visit type (in-person, video, audio only), age, race, ethnicity, rural/urban (per zip code by Federal Office of Rural Health Policy), distance from medical facility, insurance, and Digital Divide Index (DDI; incorporates technology/internet access, age, disability, and educational attainment metrics by geographic area). Pandemic phases were identified based on visit dynamics. Multivariable logistic regression models were used to examine associations of these variables with successful video visit completion.

**Results:**

Data were available for 2,398,633 visits for 516,428 patients across all specialties. Among these, there were 253,880 visits from 62,172 patients seen in any oncology clinic. Dramatic increases in telehealth usage were seen during the pandemic (after 3/16/2020). In multivariable analyses, patient age [OR: 0.964, (95% CI 0.961, 0.966) P<0.0001], rural zip code [OR: 0.814 (95% CI 0.733, 0.904) P = 0.0001], Medicaid enrollment [OR: 0.464 (95% CI 0.410, 0.525) P<0.0001], Medicare enrollment [OR: 0.822 (95% CI 0.761, 0.888) P = 0.0053], higher DDI [OR: 0.903 (95% CI 0.877, 0.930) P<0.0001], distance from the facility [OR: 1.028 (95% CI 1.021, 1.035) P<0.0001], black race [OR: 0.663 (95% CI 0.584, 0.753) P<0.0001], and Asian race [OR: 1.229 (95% CI 1.022, 1.479) P<0.0001] were associated with video visit completion early in the pandemic. Factors related to video visit completion later in the pandemic and within sub-specialties of oncology were also explored.

**Conclusions:**

Patients from older age groups, those with minority backgrounds, and individuals from areas with less access to technology (high DDI) as well as those with Medicare or Medicaid insurance were less likely to use video visits. With greater experience through the pandemic, disparities were not mitigated. Further efforts are required to optimize telehealth to benefit all patients and avoid increasing disparities in care delivery.

## Introduction

During the COVID-19 pandemic, telehealth use increased around the world to provide remote options for the management of medical conditions [1]. This increase in telehealth use occurred to balance efforts to manage new and chronic medical needs of individual patients against public health imperatives such as COVID-19 transmission risk reduction, in-person visit capacity limitations, and preservation of limited stocks of medical supplies for inpatient acute care needs [2]. Broad utilization of telehealth revealed important disparities deserving of further exploration [3].

Patients with cancer constitute a unique subset of all patients with telehealth-associated needs given the demand for complex discussions regarding plan of care for new malignancies, manifold workup steps, in-person treatment administration, and long term follow up [4]. Patients with cancer also tend to be older, may require care delivery at great distance from their residence, and represent a diverse sociodemographic group [5,6]. Very early in the COVID-19 pandemic, many thought leaders offered guidance on the care of these complex patients [7–14]. This guidance was used by many departments to balance COVID-19-associated risks with cancer-associated risks using approaches that universally involved the use of telehealth. Available data suggested that older or minority patients may be less likely to access telehealth than general patient populations [3], and practitioners have noted that telemedicine has a long term role in delivery of care beyond the pandemic [15–18]. Therefore, much work is needed to understand and address factors that might limit access and worsen health inequity that is known to exist [19].

Telehealth provides patients with a means for accessing care when in-person care options are limited. However, barriers to telehealth access that may exist for patients receiving oncologic care have not been examined in detail across the oncology space to date. As policies for oncology practices are set for the future, it will be important to carefully consider the impact that telehealth policy and practice may have on equity in access to care. The COVID-19 pandemic provides a valuable opportunity to assess what these impacts might be so that appropriate countermeasures can be developed. We examined all patient visit data from a large hospital system before and during the COVID-19 pandemic with the goal of identifying factors associated with reduced telehealth access.

## Methods

### Ethics statement

This retrospective study was conducted with the approval of the University of Michigan Medicine Institutional Review Board, who waived the requirement for informed consent for analysis of deidentified data.

### Dataset construction

Data for all unique patient visits at a large academic medical center from 7/1/2019 to 12/31/2020 were retrospectively gathered, including visit date, visit type (in-person, video, audio), age, gender, race, ethnicity, rural/urban home address (per zip code assessment by Federal Office of Rural Health Policy), insurance type, Digital Divide Index (DDI), provider, and clinic. DDI, which ranges from 0 to 100, is the sum of the infrastructure/adoption (INFA) and the socioeconomic (SE) score, which were calculated as described previously [20]. Together, the SE and INFA incorporate metrics of technology and internet access, age, disability, and educational attainment within a geographic area [20]. Lower scores are seen in areas with better access to technology and higher socioeconomic status. Data were abstracted from billing and scheduling databases. Distance from patient residence to the clinic site was approximated using zip code. The initial dataset contained all encounter types, including wellness visits, post-operative visits, evaluation, and management visits. The following visit types were excluded: infusion, radiation treatment, visits from patients who had 20+ visits, and visits from patients whose zip code of residence was outside of the United States. Pandemic phases were defined as described in the results section.

### Identification of visit specialty

Patient visits that took place in oncology practices were identified initially based on the department where each visit took place. This categorization was then edited as appropriate based on provider-associated oncologic subspecialties on a provider-by-provider basis. Next, a listing of visit provider by oncologic subspecialty was used to sort visits into the following groups: radiation oncology, surgical oncology, and medical oncology. Visits included in any of these three subspecialty groups (radiation oncology, surgical oncology, or medical oncology) were included in the group “all oncology” when analyses of all oncology visits in aggregate were conducted.

### Pandemic-associated changes in practice

Prior to March 2020, it was noted that there had been an increase in COVID-19 cases in the state of Michigan. As a result of the pandemic, there were changes in practice at Michigan Medicine in Ann Arbor, MI. By early March, those in oncology specialties had been instructed to categorize “patients according to urgency of care need: 1) those with clinical problems that require urgent and in-person evaluation and treatment, 2) patients with new or ongoing health problems of lesser urgency for whom temporary deferral of care or provision of care by virtual means will be safe, and 3) patients with routine, maintenance or non-health-compromising clinical problems for whom postponement of evaluation and treatment is safe.” In this context, many oncology visits began to occur virtually to limit exposure of patients and staff to the virus while preserving personal protective equipment, which was quite limiting at the time.

### Outcome

The primary outcome of this study was video visit completion. This was considered somewhat differently in the visit level analyses vs the subsequent patient level analyses. In the visit level analysis, all visits were considered based upon their visit type. In the initial patient level analyses, patients were categorized regarding the completion of a video visit. If a video visit was completed, then they were assumed to have the capacity to complete video visits. If they never completed a video visit but did complete audio visits, then it was assumed that they did not have the capability to complete video visits because video visits were encouraged throughout the hospital system from the first days of the pandemic, and audio visits were always discouraged. We will note that patients were scheduled for audio visits in cases where the patient made staff aware of their inability to engage in a video visit at the time of scheduling. Video visits were also converted to audio visits in cases where an inability to conduct video visits was identified at the time of the patient visit. For logistic regression models, the proportion of successfully completed video visits out of the total number of video and audio visits per patient was used to evaluate associations with patient level factors.

### Statistical analysis

Initially, data were summarized on a visit level. Next, data were summarized on a patient level. Next, bivariate analyses were conducted on a visit level and a patient level. Lastly, patient level multivariable logistic regression models were used to evaluate associations of patient factors with successful video visit completion, considering the proportional representation of audio and video visits completed by a given patient. Therefore, an audio visit was considered a failed video visit, but an in-person visit was not considered to be a failed video visit in this analysis. The following pre-specified covariates were included in all multivariable models: age, gender, race, ethnicity, rural residence, Digital Divide Index (DDI), insurance plan type (Medicaid, Medicare, private insurance, other insurance), interpreter need, and distance. No variable selection was performed. Distance was taken to be the average across the patient’s visits if the distance from the visit site varied. Separate models were fit for each of the two phases of the pandemic for all of oncology and for each of the three subspecialties: surgical, radiation, and medical oncology. Wald tests were used to assess the statistical significance of each covariate in each of these models by comparing two-sided p-values to alpha = 0.05. Odds ratio estimates and 95% confidence intervals from these models were used for construction of forest plots. All analyses were performed in SAS version 9.4 (SAS Institute Inc., Cary, North Carolina).

## Results

### Pandemic phases

**Fig 1:**
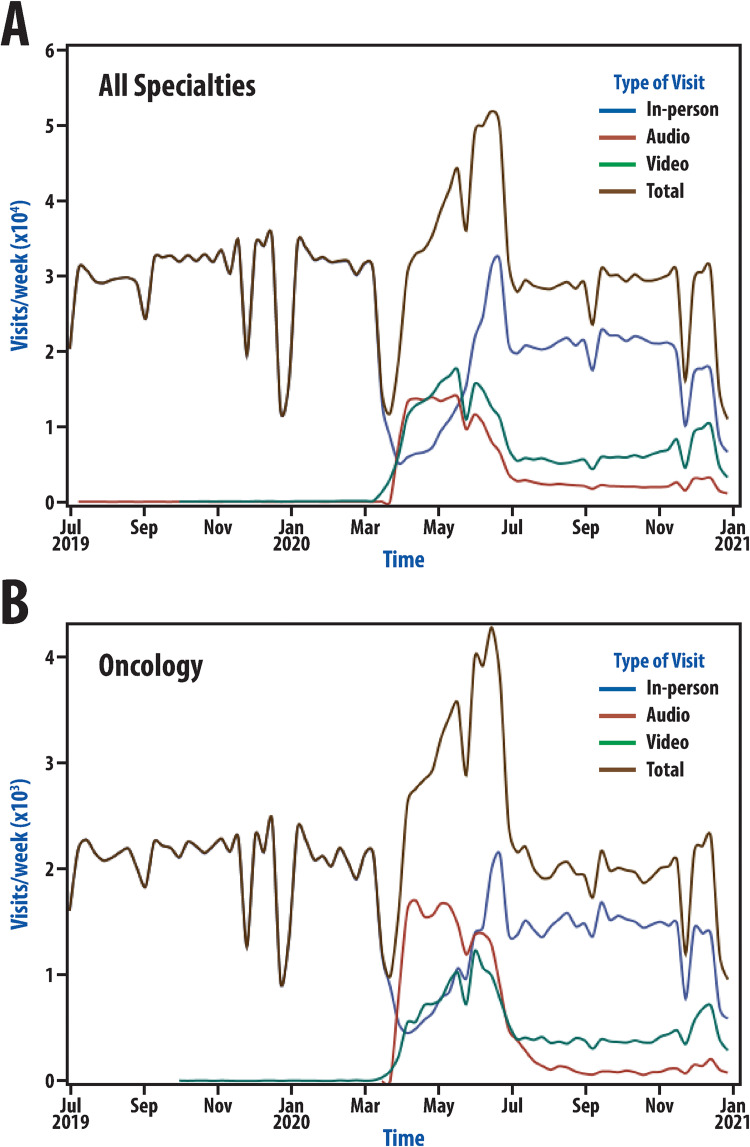
Pandemic visit dynamics at a large academic medical center. Number of visits by week from July 1, 2019 through December 31, 2020 for all specialties (A) and the subset of those visits that were held with oncology providers (B) during the COVID-19 pandemic.

**Fig 2:**
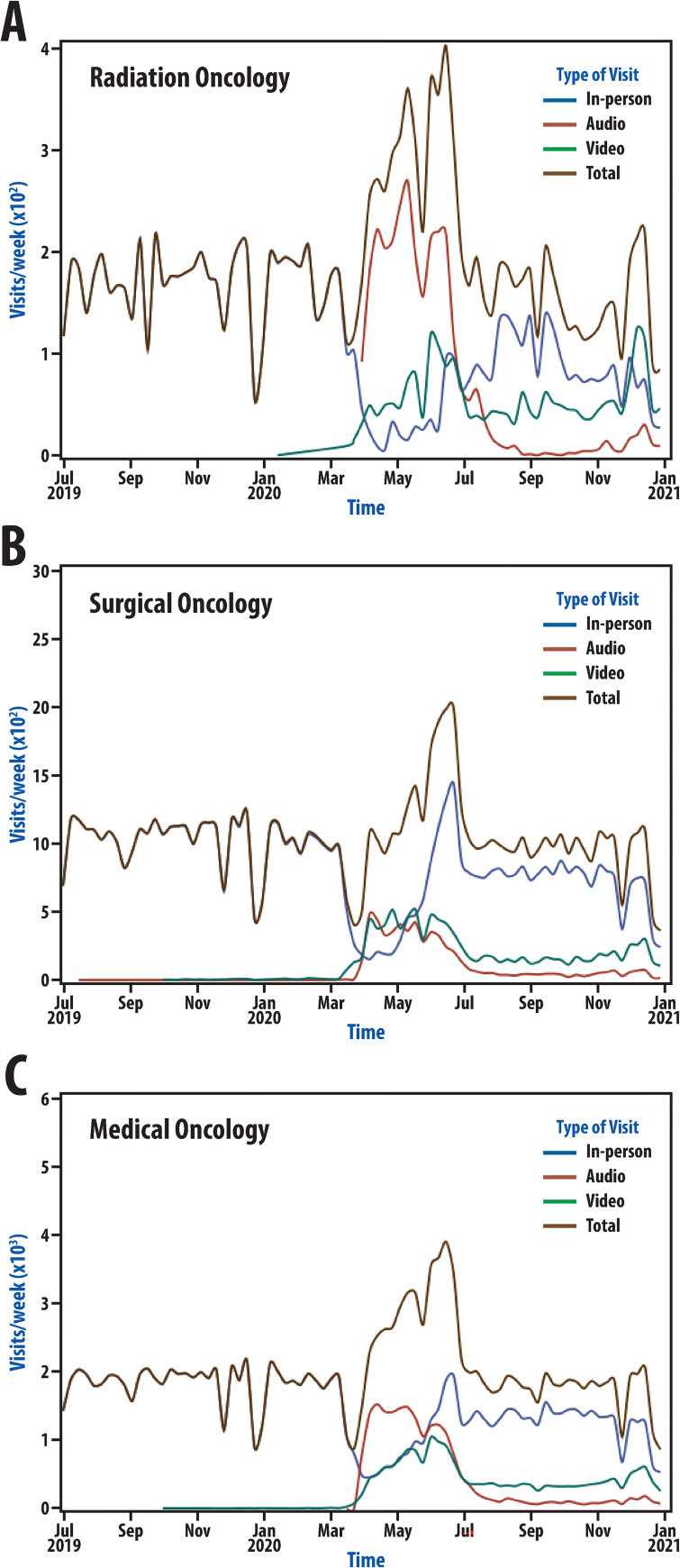
Pandemic visit dynamics among oncologic specialties. Number of visits by week from July 1, 2019 through December 31, 2020 is shown for radiation oncology (A), surgical oncology (B), and medical oncology (C) during the COVID-19 pandemic.

The full dataset included 2,398,633 visits from 7/1/2019 to 12/31/2020 in 29 departments with 4,031 providers. Visit numbers by week were graphically depicted for all visits and all of oncology to understand changes in visit distribution with time (Fig 1). Graphical representations highlight changes in visit representation, which can also be seen in Table 1 (described in detail below). There was a period pre-pandemic with little use of telehealth. With the beginning of the pandemic response, visit representation changed dramatically. This was followed by another more subtle shift in visit makeup several months after the pandemic response began; therefore, there appear to be two distinct phases of the pandemic (Fig 1). Phase 1 began on March 16, 2020 and corresponded to the first period of the pandemic response. During this phase, there was a large decrease in care delivered face-to-face with a rapid rise in telehealth use such that video and phone visits accounted for nearly two-thirds of visits. Phase 2 began on July 6, 2020; this date was selected because distinctly different telehealth usage characteristics were observed after this date compared to either Phase 1 or the pre-pandemic period. During this period, there was a persistent use of telehealth, though proportional representation changed to again favor in-person visits. There was a large decline in the proportion of visits completed by phone between Phase 1 and Phase 2 while video visits made up approximately one in five visits during this period. Visit patterns in radiation oncology, medical oncology, and surgical oncology were similar (Fig 2).

**Table 1:** Details of visit characteristics during different phases of the COVID-19 pandemic.

Specialty by Phase	Video	Phone	In-person	Total
	N (%)	N (%)	N (%)	N
**Pre-pandemic Phase**	–	–	–	–
All specialties	1,658 (0.15%)	114 (0.01%)	1,111,130 (99.84%)	1,112,902
All Oncology	276 (0.24%)	15 (0.01%)	112,973 (99.74%)	113,264
Radiation Oncology	1 (0.01%)	0 (0.00%)	6,863 (99.99%)	6,864
Medical Oncology	85 (0.12%)	0 (0.00%)	68,316 (99.88%)	68,401
Surgical Oncology	190 (0.50%)	15 (0.04%)	37,794 (99.46%)	37,999
**Phase I**	–	–	–	–
All specialties	184,791 (32.37%)	153,326 (26.86%)	232,819 (40.78%)	570,936
All Oncology	16,461 (24.86%)	23,904 (36.10%)	25,853 (39.04%)	66,218
Radiation Oncology	1,006 (22.39%)	2,675 (59.52%)	813 (18.09%)	4,494
Medical Oncology	9,729 (23.00%)	16,697 (39.47%)	15,879 (37.53%)	42,305
Surgical Oncology	5,726 (29.49%)	4,532 (23.34%)	9,161 (47.18%)	19,419
**Phase 2**	–	–	–	–
All specialties	159,609 (22.33%)	57,933 (8.10%)	497,253 (69.57%)	714,795
All Oncology	15,331 (20.62%)	4,672 (6.28%)	54,395 (73.10%)	74,398
Radiation Oncology	1,376 (32.36%)	372 (8.75%)	2,504 (58.89%)	4,252
Medical Oncology	9,615 (21.03%)	3,076 (6.73%)	33,036 (72.25%)	45,727
Surgical Oncology	4,340 (17.77%)	1,224 (5.01%)	18,855 (77.21%)	24,419
**All Phases**	–	–	–	–
All specialties	346,058 (14.43%)	211,373 (8.81%)	1,841,202 (76.76%)	2,398,633
All Oncology	32,068 (12.63%)	28,591 (11.26%)	193,221 (76.11%)	253,880
Radiation Oncology	2,383 (15.27%)	3,047 (19.52%)	10,180 (65.21%)	15,610
Medical Oncology	19,429 (12.42%)	19,773 (12.64%)	117,231 (74.94%)	156,433
Surgical Oncology	10,256 (12.53%)	5,771 (7.05%)	65,810 (80.42%)	81,837

Definitions: Pre-pandemic: July 1, 2019 –March 15, 2020; Phase 1: March 16, 2020 –July 5, 2020; Phase 2: July 6, 2020 –December 31, 2020.

Abbreviations: N–number.

### Visit level data summary

Visit level summary statistics are presented in Table 1 for the pre-pandemic period as well as pandemic Phase 1 and pandemic Phase 2. Over all periods, there were 253,880 oncology visits. Of these, 193,221 (76.1%), 32,068 (12.6%), and 28,591 (11.3%) were in-person, video, and audio, respectfully (Table 1). In radiation oncology, 10,180 (65.2%), 2,383 (15.3%), and 3,047 (19.5%) visits were in-person, video, and audio, respectfully. In surgical oncology, 65,810 (80.42%), 10,256 (12.53%), and 5,771 (7.05%) visits were in-person, video, and audio, respectfully. In medical oncology, 117,231 (74.94%), 19,429 (12.42%), and 19,773 (12.64%) visits were in-person, video, and audio, respectfully. Significant variation in visit type distribution was noted across phases and specialties (S1 Appendix).

### Patient level data summary

Patient level summary statistics with demographic and patient characteristics are provided for all of oncology (Table 2) and for the oncologic specialties, including, radiation, surgical, and medical oncology during pandemic Phase 1 and Phase 2 (S1 appendix). Patients seen in any oncologic specialty clinic had a median age of 61, were 50.84% female, and 87.21% Caucasian. In the presentation shown in Table 2, a patient was considered a video visit user if they completed at least one video visit. A patient was considered a phone visit user if they completed at least one phone but no video visits. A patient was considered a non-telehealth user if they did not complete any video or audio visits (only in-person visits). P values for bivariate patient level analyses are presented, and gender, age, race, ethnicity, need for interpreter, insurance (Medicaid, Medicare, private insurance, and other insurance), and residency impacted the distribution of patients among user groups (Table 2).

**Table 2:** Characteristics of patients seen in any oncology specialty (N = 62,172) during the COVID-19 pandemic (Phase 1 and Phase 2) using each visit type.

Variable	Video visit users^e^	Phone visit users^f^	Non-telehealth users^g^	p-value
	N = 20,619	N = 16,110	N = 25,443	
**Gender**	–	–	–	<0.0001
Female	10,740 (34.24%)	7,623 (24.31%)	13,000 (41.45%)	
Male	9,876 (32.06%)	8,487 (27.55%)	12,441 (40.39%)	
**Age** ^a^	53.94	63.16	55.64	<0.0001
**Race**	–	–	–	<0.0001
White or Caucasian	17,667 (33.77%)	13,622 (26.04%)	21,026 (40.19%)	
Black or African-American	1,206 (28.12%)	1,307 (30.47%)	1,776 (41.41%)	
Asian	636 (37.77%)	309 (18.35%)	739 (43.88%)	
All Others ^b^	501 (30.91%)	415 (25.60%)	705 (43.49%)	
**Ethnicity**	–	–	–	0.0175
Non-Hispanic	19,121 (33.48%)	14,922 (26.12%)	23,075 (40.40%)	
Hispanic	441 (32.72%)	315 (23.37%)	592 (43.92%)	
**Needed Interpreter**	–	–	–	<0.0001
Yes	143 (21.38%)	184 (27.50%)	342 (51.12%)	
No	20,306 (33.26%)	15,859 (25.97%)	24,890 (40.77%)	
**Insurance Plan**	–	–	–	<0.0001
Medicaid	1,420 (29.28%)	1,216 (25.07%)	2,214 (45.65%)	
Medicare	7,393 (26.78%)	9,304 (33.70%)	10,913 (39.53%)	
Private	10,792 (40.34%)	5,157 (19.28%)	10,804 (40.38%)	
Other	154 (29.17%)	132 (25.00%)	242 (45.83%)	
**Rural residence** ^c^	–	–	–	<0.0001
Yes	3,362 (32.43%)	2,858 (27.57%)	4,148 (40.01%)	
No	16,871 (34.21%)	12,594 (25.54%)	19,851 (40.25%)	
**Broadband access** ^**a**^^,^^**c**^	82.37%	81.17%	81.61%	<0.0001
**Below poverty threshold** ^**a**^^,^^**c**^	13.02%	13.75%	13.66%	<0.0001
**Income** ^**d**^	66,729.53	63,446.99	64,438.39	<0.0001
**Digital Divide Index** ^**a**^	28.39	30.06	29.42	<0.0001

^a^Mean.

^b^All others–specified as “American Indian”, “Alaska Native”, or “Other”.

^c^Mean percentage of households by zip code.

^d^Mean of households by zip code in dollars.

^e^Video visit users–patients who completed at least one video visit.

^f^Phone visit users–patients who completed at least one audio visit but no video visits.

^g^Non-telehealth users–patients who completed no audio or video visits.

### Patient level multivariable analyses of factors related to video visit completion

**Fig 3:**
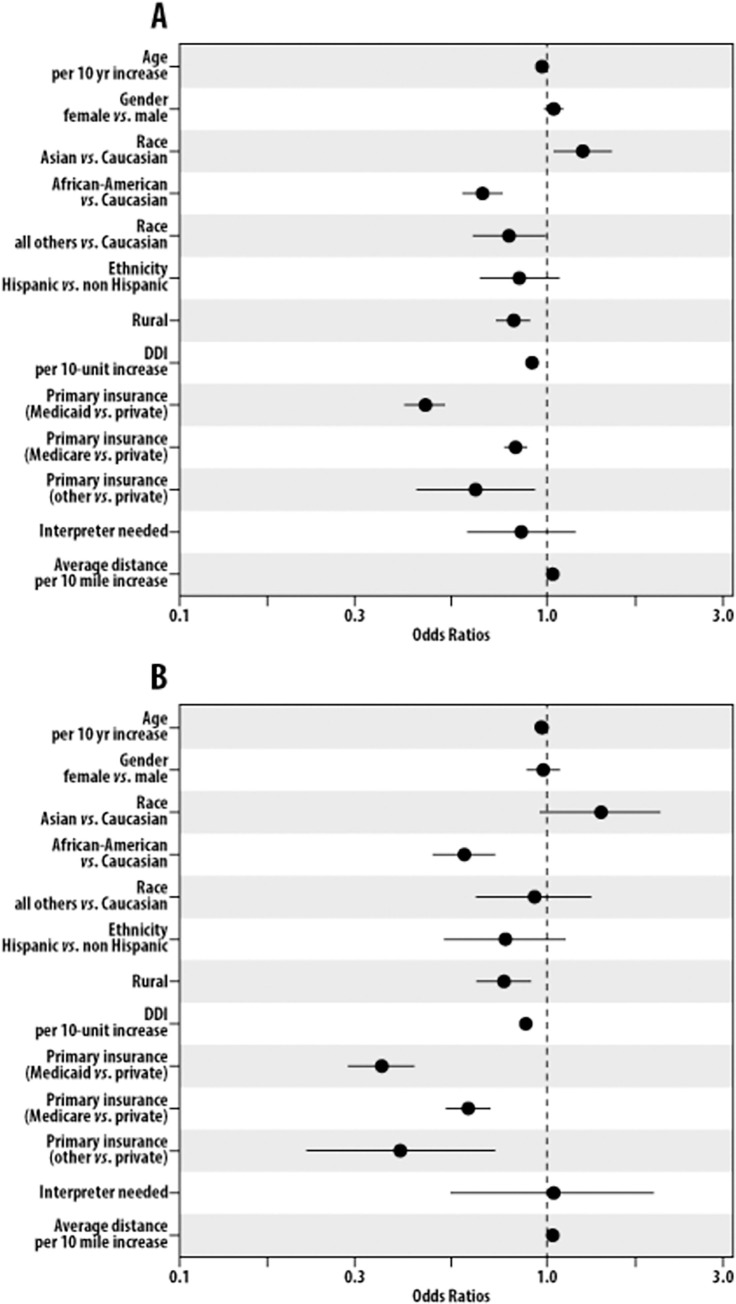
Predictors of video visit completion. Forest plots displaying predictors of video visit completion in all of oncology during Phase 1 (A) and Phase 2 (B) of the COVID-19 pandemic.

Having established the phases of the pandemic and generally assessed in a simple patient level analysis factors related to visit type distributions, we next used multivariable analyses of patient level data to examine factors that were associated with completion of video visits in hopes of identifying potential barriers to telehealth access during each phase across oncology and within radiation oncology, surgical oncology, and medical oncology (S1 Appendix). When Phase 1 visits across oncology were considered, older age [OR: 0.964, (95% CI 0.961, 0.966) P<0.0001], black race [OR: 0.663 (95% CI 0.584, 0.753) P<0.0001], rural zip code [OR: 0.814 (95% CI 0.733, 0.904) P = 0.0001], Medicaid enrollment [OR: 0.464 (95% CI 0.410, 0.525) P<0.0001], Medicare enrollment [OR: 0.822 (95% CI 0.761, 0.888) P = 0.0053] and higher DDI [OR: 0.903 (95% CI 0.877, 0.930) P<0.0001] were associated with lack of video visit completion, while increased distance from the facility [OR: 1.028 (95% CI 1.021, 1.035) P<0.0001] and Asian race [OR: 1.229 (95% CI 1.022, 1.479) P<0.0001] were associated with video visit completion (Fig 3; S1 Appendix). Gender, ethnicity, and interpreter usage did not impact ability to complete video visits. Despite the difference between the pandemic telehealth phases with regard to visit type distribution, similar patterns of association were seen for all of oncology during Phase 2 of the pandemic to those seen in Phase 1 save that Medicare enrollment was no longer associated with lack of video visit completion (Fig 3; S1 Appendix).

After examining predictors of video visit completion among all oncology visits in aggregate, we next explored similar analyses for each oncologic subspecialty (radiation oncology, surgical oncology, and medical oncology). Generally, similar trends were noted in each of the subspecialties to the findings from the analysis of all oncology visits together. In radiation oncology, we noted that older age [OR: 0.980 (95% CI 0.970, 0.990) P<0.0001], Medicaid enrollment [OR: 0.366 (95% CI 0.217, 0.617) P = 0.0097], and higher DDI [OR: 0.874 (95% CI 0.803, 0.952) P = 0.0020] were associated with lack of video visit completion (S1 Appendix). Increasing distance from the facility (P = 0.0065) was protective of a patient’s ability to complete video visits. Findings were similar in Phase 2 with age, DDI, Medicaid enrollment, and distance from facility significantly associated with video visit completion. However, females were less likely to complete video visits [OR: 0.620 (95% CI 0.462, 0.832) P = 0.0015]. The impact of race was less notable in radiation oncology than seen when all oncology patients were examined in aggregate (S1 Appendix). Findings from surgical oncology and medical oncology were similar to those seen in radiation oncology, with increased age, Medicaid enrollment, and higher DDI associated with lack of video visit completion (S1 Appendix). Distance remained protective in surgical and medical oncology for both phases of the pandemic. Race was significant for medical oncology in both phases but not for surgical oncology or radiation oncology in either phase. Details are included in the (S1 Appendix).

## Discussion

Through analysis of all patient visits in a large academic medical center, we noted dramatic shifts in telehealth utilization during the early days of the COVID-19 pandemic. Among those seeking cancer care, we found that older individuals, African Americans, those utilizing Medicaid, and those from areas with higher DDI were less likely to complete video visits. These factors support a number of considerations for future telehealth applications across oncologic specialties. First, older individuals who make up the bulk of oncology patients may need additional help in accessing telehealth. Second, racial inequities in cancer care may be exacerbated through telehealth, suggesting the need for effective means to mitigate telehealth-related disparities in the post-COVID-19 world. Third, those from rural areas may require additional support in order to access telehealth resources. Fourth, Digital Divide Index is related to telehealth use, suggesting that investments in broader access to the internet and quality education may have important positive implications for oncologic care access. There is great interest in preserving telehealth advances to facilitate care delivery in the future. Our analyses supports the findings of others who have suggested that addressing barriers and inequality concerns will be critical to ensure full access for all oncology patients [15,18,21].

### Visit dynamics during the pandemic

Institutional guidance borne out of efforts to protect patients and providers from COVID-19 led to initial shifts in visit types during the pandemic in the general patient population and among patients seen by oncologic specialties at our institution. Others have shown dramatic shifts in visit type in response to COVID-19 as visit dynamics have been reported extensively in the growing COVID-19 pandemic literature [22,23]. Across the US and around the world, guidance came at slightly different timepoints in the pandemic, accounting for locoregional variation in viral case numbers and resources. While generalizations across the United States with regard to the precise date selections that we have made in Fig 1 are not possible, it is likely that review of data from many institutions in the United States would demonstrate large shifts in visit composition at timepoints specific to individual healthcare systems as they responded to the needs of their patient populations. Though large shifts occurred universally, the nature of these shifts was likely dictated by many features of individual institutions such as technological resources and telehealth expertise as well as other contextual factors beyond individual health systems. Generally, the timing seen at our institution in March 2020 is consistent with timing reported at other institutions [23].

Outside of health system and institutional decisions made in the context of an understanding of the pandemic locally, there are other factors that should be considered at different levels that impact whether an individual patient might complete a video visit. These might vary across the pandemic period. Payor practices have guided telehealth use and enabled dramatic increases in telehealth use [24], highlighting the fact that any approach to telehealth in oncology will require collaboration between medical institutions and payors [25]. Additionally, patients can be taught, so it is likely that some patients might have gained the capacity to complete telehealth visits during the pandemic. Providers and institutions might also have varied skill sets and resources to enable patients’ efforts to engage in telehealth. Patient and provider skills as well as payor guidance are all in flux along with the pace of the pandemic itself. It will be important for institutions, payors, providers, and patients themselves to have a voice in the process of refining telehealth as a component of cancer care delivery. Additionally, it will be important to consider all of the many factors that impact telehealth use in future studies designed to optimize its fair and ethical application.

### Predictors of telehealth access in oncology

#### Older individuals

As age increased, patients were less likely to use video visits. It is known that many older adults lack access to the technology and expertise required to participate fully in telehealth [26]. Younger patients with cancer are more likely to prefer telehealth visits than older patients with cancer [27]. Outside of oncology, analyses across primary and specialty practices have shown that older adults are less likely to participate in video visits [22,28]. A study of patients at another cancer center similarly found that older individuals were less likely to use video visits [29]. In a previous non-oncology focused analysis from our institution during a shorter time frame than that outlined in our manuscript, the mean ages for video visit users, phone visit users, and non-telehealth users were 42, 56, and 41, respectively [28]. However, in our analysis, the mean ages for video visit users, phone visit users, and non-telehealth users were 54, 63, and 56, respectively. Because the oncology patient population is older than the general population, any age-related burdens or barriers will negatively impact patients with cancer to a greater degree. Patients seen in radiation oncology were the oldest of the three oncologic specialties, suggesting that age-related telehealth access difficulties may be most challenging in radiation oncology, among oncologic providers. Our work agrees with the findings of others who have noted that special attention must be paid to older patients to facilitate access to telehealth [30,31].

#### Race

People of color have been shown to have greater difficulty accessing telehealth in multiple prior studies [31–33]. In the data described in this report, the impact of race on video visit completion varied somewhat depending on whether the analysis included all oncology patients or various subspecialties that make up oncology. It is likely that these differences are the result of variable patient numbers and power to obtain statistical significance given the magnitude of odds ratios shown in Fig 3 (also S1 Appendix). Our findings in the aggregate analysis of all oncology visits resulted in similar findings to those of Shao et al who studied a population from an NCI designated cancer center in the state of Alabama (in a population with greater minority representation than that seen in our study population) and found that patients of color were less likely to use video visits [29]. This supports the concern that telehealth might exacerbate existing inequities in healthcare and cancer care specifically if countermeasures are not developed [19].

#### Gender

Some have found an impact of gender on video visit completion, specifically male gender [29]. We did not see an impact of gender in our aggregate analysis of oncology visits. However, an effect of gender was noted variably across pandemic phases and specialties such that it is difficult to draw strong conclusions on the impact of gender using our data (S1 Appendix). Females were less likely to complete video visits in Phase 2 within radiation oncology and Phase 1 within surgical oncology. Further exploration of the impact of gender is warranted as efforts to optimize telehealth access proceed.

#### DDI

The Digital Divide Index predicted video visit completion in the present study. This metric is complex in that it incorporates access to broadband internet, computing devices, download speed, upload speed, age, education, poverty rate, and disability within a geographical area. It was highly significant in all analyses in models containing age. Therefore, it is likely that the effect is driven by other components of the score. Many of the components relate to available infrastructure. Further enhancing access to broadband is likely to increase access to telehealth. Likewise, the significance of DDI may also point to the potential for telehealth to worsen educational attainment-related disparities in health outcomes in oncology. Patients from areas with lower income have been shown to be less likely to complete video visits by others [29]. Regional socioeconomic status and DDI are also clearly quite closely entwined. DDI could be easily adapted to guide interventions to address barriers to telehealth access.

#### Insurance

In our study, those with Medicare or Medicaid were less likely than those with private insurance to engage in video visits. This is similar to the results of an examination of patients at another cancer center, where patients with public insurance were less likely to engage in video visits [29].

#### Rural zip code

Those from rural zip codes were less likely to complete video visits. These findings generally parallel findings of previous research on factors that are related to lower rates of patient portal usage [31]. Efforts to improve oncologic care delivery through the use of telehealth are being studied; these ongoing efforts will need to carefully account for telehealth access-related concerns of rural patients with cancer [34].

#### Distance

Increasing distance from the medical facility was associated with increased ability to complete video visits. This was somewhat unexpected and may have been a product of a greater perceived incentive in the form of avoiding a lengthy trip to receive care. For example, those patients at greater distance from healthcare facilities might be more likely to organize approaches to take part in telehealth and to plan ahead prior to virtual visits with providers in hopes of avoiding the extra cost and effort required to attend visits in person. It is also possible that individuals living in remote areas were more likely to have engaged in video visits with another system prior to their encounter in our system. These previous visits might then have served as practice sessions, allowing patients time to train themselves prior to their initial encounters with our center. A better understanding of factors that motivate patient engagement with telehealth will be important to the further adoption of telehealth in the future.

### The future of telehealth in oncologic care

Dramatic changes in oncology department operation occurred during the COVID-19 pandemic [13,35]. Great efforts were undertaken in radiation oncology practices toward reducing the number of patients under treatment [18,36]. Changes were suggested for infusion protocols in medical oncology practices [13]. Surgeons were encouraged to consider non-operative management where possible [14]. As oncologic care has reached a new steady state, physicians express high levels of satisfaction with the use of telehealth [16,37,38]. These providers report that they will continue offering telehealth visits, and practice guidelines for telehealth have been developed [15,39–41].

Despite this, support is not uniform; skeptics caution that telehealth may lead to lower quality patient care [18] or exacerbate care disparities. Additionally, patients with positive impressions of telehealth still recognized the importance of in-person physical exams for detection of cancer recurrence [42]. This dialog supports discussion of approaches to balance in-person and telehealth visits to achieve patient and provider goals. Some have envisioned ways that telehealth could help reduce disparities [17,21]. As elements of telehealth continue beyond the COVID-19 pandemic in the area of on treatment monitoring in radiation oncology, long term follow up in all specialties, and in selected pre-treatment settings [17,25,43], developing models of telehealth that balance in-person and remote patient care priorities while addressing disparities will be necessary prerequisites for quality care delivery across oncologic specialties.

### Were improvements observed over the course of the pandemic?

At our institution, two distinct pandemic phases were noted. Though many have examined telehealth during the pandemic and found inequities early in the pandemic, we show that these inequities persisted beyond the initial phase of the pandemic. There is little difference in significance of many key features from Phase 1 and Phase 2. Specifically, the odds ratios for the impact of race, DDI, Medicaid enrollment status, and age are largely unchanged between Phase 1 and Phase 2. This illustrates that there is still much work to be done to improve access to telehealth-based oncologic care.

### Study limitations

This study uses data from a single large academic institution in a single area of the United States. Therefore, the dynamics of visit type distributions may not be generalizable to all clinical settings but may apply to similarly sized academic and non-academic centers with large catchment areas. It is important to note that people who were not Caucasian made up 18.87% of the population studied. Further study in more diverse populations will be helpful in the future. We recognize that there may be competing reasons why an individual might not complete a video visit. For example, the nature of their care might necessitate that they be seen in person for clinical reasons. It is also not possible to assess the impact of a family member with appropriate skills or other resources that might help patients complete virtual visits. Because our institution was strongly encouraging video visits whenever possible, we feel that it is reasonable to make the assumption that the great majority of patients would have been subject to a request to complete a video visit during the relevant period. It is likely that many other factors would bias toward not away from the null hypothesis when considering the impact of other factors.

## Conclusions

The COVID-19 pandemic provides a valuable opportunity to study telehealth and more specifically barriers to access and disparities. Patients, providers, and regulators have seen benefits from telehealth, and as a result, features of telehealth-based care delivery will be preserved in the future. Therefore, it is imperative that steps be taken to ensure access to high quality telehealth for those less likely able to fully participate, including older patients, those from rural areas, those with lower socioeconomic status, and those with lower levels of infrastructural and past educational support.

## Supporting information

S1 Appendix Analysis of factors related to video visit completion in each oncologic specialty alone (radiation oncology, surgical oncology, and medical oncology) and in the aggregate of all of oncology. Data are presented in figure and tabular form.
(DOCX)
