# Supplementary material for: Age, race, insurance type, and digital divide index are associated with video visit completion for patients seen for oncologic care in a large hospital system during the COVID-19 pandemic
Source: PLoS One. 2022 Nov 17;17(11):e0277617. doi: 10.1371/journal.pone.0277617 (PMC9671352; doi:10.1371/journal.pone.0277617)
Supplement: S1 Appendix — Data are presented in figure and tabular form. (DOCX) [file pone.0277617.s001.docx]

**S1 Appendix**

Age, race, insurance type, and digital divide index are associated with video visit completion for patients seen for oncologic care in a large hospital system during the COVID-19 pandemic

Matthew M. Cousins^1^, Monica Van Til^1,2^, Emma Steppe^3^, Sophia Ng^3^, Chandy Ellimoottil^3,4^, Yilun Sun^1,2^, Matthew Schipper^1,2^, and Joseph R. Evans^1^*

**^1^**Department of Radiation Oncology, University of Michigan, Ann Arbor, Michigan, United States of America

^2^Department of Biostatistics, University of Michigan, Ann Arbor, Michigan, United States of America

^3^Institute for Healthcare Policy and Innovation, University of Michigan, Ann Arbor, Michigan, United States of America

^4^Department of Urology, University of Michigan, Ann Arbor, Michigan, United States of America

Table of Contents:

| *Page* | *Items* |
| --- | --- |
| 4 ……….. | S1 Fig. Predictors of video visit completion in radiation oncology clinic. Forest plots displaying predictors of video visit completion in radiation oncology clinic during Phase 1 (A) and Phase 2 (B) of the pandemic. |
| 5 ……….. | S2 Fig. Predictors of video visit completion in surgical oncology clinic. Forest plots displaying predictors of video visit completion in surgical oncology clinic during Phase 1 (A) and Phase 2 (B) of the pandemic. |
| 6 ……….. | S3 Fig. Predictors of video visit completion in medical oncology clinic. Forest plots displaying predictors of video visit completion in medical oncology clinic during Phase 1 (A) and Phase 2 (B) of the pandemic. |
| 7 ……….. | S1 Table. Statistical analysis of number of visits apportioned to video, phone, and in-person across oncologic specialty by pandemic phase. P-values are derived from chi-square testing on visit level data (Table 1). |
| 8 ……….. | S2 Table. Characteristics of patients seen in radiation oncology (N=6,543) using each visit type. |
| 9 ……….. | S3 Table. Characteristics of patients seen in surgical oncology (N=28,029) using each visit type. |
| 10……….. | S4 Table. Characteristics of patients seen in medical oncology (N=37,581) using each visit type. |
| 11 ………. | S5 Table. Logistic regression models for all of oncology during Phase 1 of the COVID-19 pandemic (N=15,719 patients). |
| 11 ………. | S6 Table. Odds ratios for all of oncology during Phase 1 of the COVID-19 pandemic (N=15,719 patients). |
| 12 ………. | S7 Table. Logistic regression models for radiation oncology during Phase 1 of the COVID-19 pandemic (N=2,648 patients). |
| 12 ………. | S8 Table. Odds ratios for radiation oncology during Phase 1 of the COVID-19 pandemic (N=2,648 patients). |
| 13 ………. | S9 Table. Logistic regression models for surgical oncology during Phase 1 of the COVID-19 pandemic (N=2,026 patients). |
| 13 ………. | S10 Table. Odds ratios for surgical oncology during Phase 1 of the COVID-19 pandemic (N=2,026 patients). |
| 14 ………. | S11 Table. Logistic regression models for medical oncology during Phase 1 of the COVID-19 pandemic (N=12,222 patients). |
| 14 ………. | S12 Table. Odds ratios for medical oncology during Phase 1 of the COVID-19 pandemic (N=12,222 patients). |
| 15 ………. | S13 Table. Logistic regression models for all of oncology during Phase 2 of the COVID-19 pandemic (N=6,716 patients). |
| 15 ………. | S14 Table. Odds ratios for all of oncology during Phase 2 of the COVID-19 pandemic (N=6,716 patients). |
| 16 ………. | S15 Table. Logistic regression models for radiation oncology during Phase 2 of the COVID-19 pandemic (N=1,124 patients). |
| 16 ………. | S16 Table. Odds ratios for radiation oncology during Phase 2 of the COVID-19 pandemic (N=1,124 patients). |
| 17 ………. | S17 Table. Logistic regression models for surgical oncology during Phase 2 of the COVID-19 pandemic (N=752 patients). |
| 17 ………. | S18 Table. Odds ratios for surgical oncology during Phase 2 of the COVID-19 pandemic (N=752 patients). |
| 18 ………. | S19 Table. Logistic regression models for medical oncology during Phase 2 of the COVID-19 pandemic (N=5,365 patients). |
| 18 ………. | S20 Table. Odds ratios for medical oncology during Phase 2 of the COVID-19 pandemic (N=5,365 patients). |

Table Footnotes:

^a^Mean; ^b^All others – specified as “American Indian”, “Alaska Native”, or “Other”; ^c^Mean percentage of households by zip code; ^d^Mean of households by zip code

Abbreviations: DDI – Digital Divide Index; White – White or Caucasian

**S1 Fig**. **Predictors of video visit completion in radiation oncology clinic.** Forest plots displaying predictors of video visit completion in radiation oncology during (A) Phase 1 and (B) Phase 2 of the pandemic.


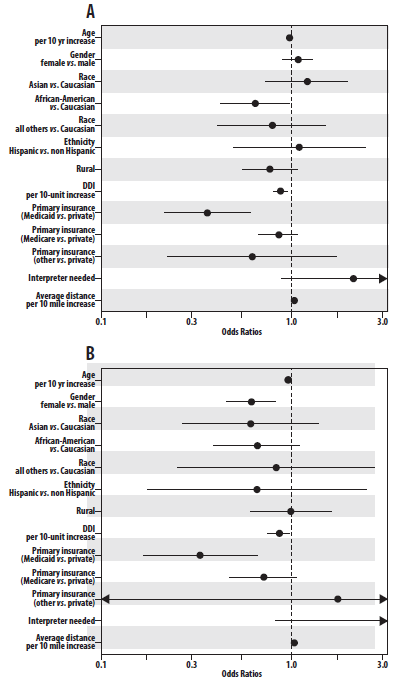


**S2 Fig**. **Predictors of video visit completion in surgical oncology clinic.** Forest plots displaying predictors of video visit completion in radiation oncology during (A) Phase 1 and (B) Phase 2 of the pandemic.


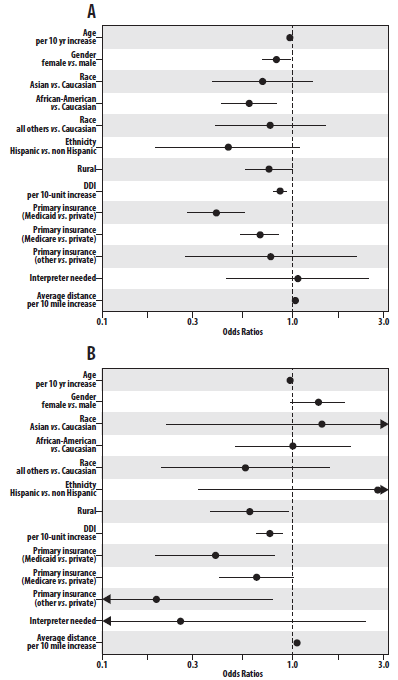


**S3 Fig**. **Predictors of video visit completion in medical oncology clinic.** Forest plots displaying predictors of video visit completion in radiation oncology during (A) Phase 1 and (B) Phase 2 of the pandemic.


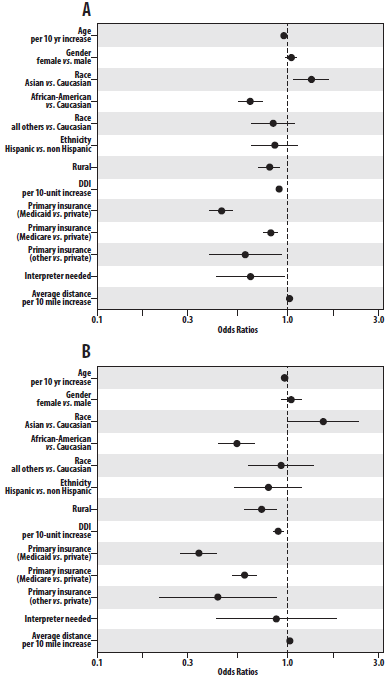


**S1 Table.** Statistical analysis of number of visits apportioned to video, phone, and in-person across oncologic specialty by pandemic phase. P-values are derived from chi-square testing on visit level data (Table 1).

| **Specialty by Phase** | **Video**  **N (%)** | **Phone**  **N (%)** | **In-person**  **N (%)** | **p-value** |
| --- | --- | --- | --- | --- |
| Pre-pandemic Phase | – | – | – | <0.0001 |
| Radiation Oncology | 1 (0.01%) | 0 (0.00%) | 6,863 (99.99%) | – |
| Medical Oncology | 85 (0.12%) | 0 (0.00%) | 68,316 (99.88%) | – |
| Surgical Oncology | 190 (0.50%) | 15 (0.04%) | 37,794 (99.46%) | – |
| Phase I | – | – | – | <0.0001 |
| Radiation Oncology | 1,006 (22.39%) | 2,675 (59.52%) | 813 (18.09%) | – |
| Medical Oncology | 9,729 (23.00%) | 16,697 (39.47%) | 15,879 (37.53%) | – |
| Surgical Oncology | 5,726 (29.49%) | 4,532 (23.34%) | 9,161 (47.18%) | – |
| Phase 2 | – | – | – | <0.0001 |
| Radiation Oncology | 1,376 (32.36%) | 372 (8.75%) | 2,504 (58.89%) | – |
| Medical Oncology | 9,615 (21.03%) | 3,076 (6.73%) | 33,036 (72.25%) | – |
| Surgical Oncology | 4,340 (17.77%) | 1,224 (5.01%) | 18,855 (77.21%) | – |
| All Phases | – | – | – | <0.0001 |
| Radiation Oncology | 2,383 (15.27%) | 3,047 (19.52%) | 10,180 (65.21%) | – |
| Medical Oncology | 19,429 (12.42%) | 19,773 (12.64%) | 117,231 (74.94%) | – |
| Surgical Oncology | 10,256 (12.53%) | 5,771 (7.05%) | 65,810 (80.42%) | – |

Definitions: Pre-pandemic: July 1, 2019 – March 15, 2020; Phase 1: March 16, 2020 – July 5, 2020; Phase 2: July 6, 2020 – December 31, 2020

Abbreviations: N – number

**S2 Table.** Characteristics of patients seen in radiation oncology (N=6,543) using each visit type.

| **Variable** | **Video visit users**  **N=2,082** | **Phone visit users**  **N=2,560** | **Non-telehealth users**  **N=1,901** | **p-value** |
| --- | --- | --- | --- | --- |
| **Gender** | – | – | – | 0.0210 |
| Female | 829 (32.14%) | 960 (37.22%) | 790 (30.63%) | – |
| Male | 1,253 (31.61%) | 1,600 (40.36%) | 1,111 (28.03%) | – |
| **Age** ^a^ | 62.18 | 65.92 | 63.41 | <0.0001 |
| **Race** | – | – | – | <0.0001 |
| White or Caucasian | 1,820 (32.45%) | 2,222 (39.61%) | 1,567 (27.94%) | – |
| Black or African-American | 104 (22.76%) | 183 (40.04%) | 170 (37.20%) | – |
| Asian | 71 (37.37%) | 61 (32.11%) | 58 (30.53%) | – |
| All Others ^b^ | 40 (30.53%) | 49 (37.40%) | 42 (32.06%) | – |
| **Ethnicity** | – | – | – | 0.2793 |
| Non-Hispanic | 1,970 (32.12%) | 2,411 (39.31%) | 1,752 (28.57%) | – |
| Hispanic | 19 (23.75%) | 35 (43.75%) | 26 (32.50%) | – |
| **Needed Interpreter** | – | – | – | 0.0839 |
| Yes | 20 (33.33%) | 16 (26.67%) | 24 (40.00%) | – |
| No | 2,052 (31.76%) | 2,538 (39.28%) | 1,871 (28.96%) | – |
| **Primary Plan** | – | – | – | <0.0001 |
| Medicaid | 74 (22.77%) | 138 (42.46%) | 113 (34.77%) | – |
| Medicare | 1,002 (28.27%) | 1,557 (43.93%) | 985 (27.79%) | – |
| Private | 955 (37.64%) | 825 (32.52%) | 757 (29.84%) | – |
| Other | 15 (34.09%) | 15 (34.09%) | 14 (31.82%) | – |
| **Rural residence ^c^** | – | – | – | 0.0130 |
| Yes | 296 (33.04%) | 380 (42.41%) | 220 (24.55%) | – |
| No | 1,746 (32.28%) | 2,087 (38.58%) | 1,576 (29.14%) | – |
| **Broadband access ^a,c^** | 83.15% | 81.67% | 82.38% | <0.0001 |
| **Below poverty threshold ^a,c^** | 12.75% | 13.51% | 12.84% | 0.0590 |
| **Income ^d^** | 68,957.93 | 64,097.47 | 66,656.15 | <0.0001 |
| **Digital Divide Index ^d^** | 27.47 | 29.26 | 28.21 | <0.0001 |

**S3 Table.** Characteristics of patients seen in surgical oncology (N=28,029) using each visit type.

| **Variable** | **Video visit users**  **N=7,768** | **Phone visit users**  **N=4,555** | **Non-telehealth users**  **N=15,706** | **p-value** |
| --- | --- | --- | --- | --- |
| **Gender** | – | – | – | <0.0001 |
| Female | 3,474 (26.27%) | 1,903 (14.39%) | 7,845 (59.33%) | – |
| Male | 4,294 (29.00%) | 2,652 (17.91%) | 7,861 (53.09%) | – |
| **Age** ^a^ | 58.29 | 64.13 | 60.28 | <0.0001 |
| **Race** | – | – | – | <0.0001 |
| White or Caucasian | 6,759 (28.38%) | 3,826 (16.06%) | 13,233 (55.56%) | – |
| Black or African-American | 394 (22.87%) | 356 (20.66%) | 973 (56.47%) | – |
| Asian | 185 (26.09%) | 95 (13.40%) | 429 (60.51%) | – |
| All Others ^b^ | 164 (24.55%) | 113 (16.92%) | 391 (58.53%) | – |
| **Ethnicity** | – | – | – | 0.1805 |
| Non-Hispanic | 7,195 (27.95%) | 4,192 (16.29%) | 14,353 (55.76%) | – |
| Hispanic | 130 (24.44%) | 87 (16.35%) | 315 (59.21%) | – |
| **Needed Interpreter** | – | – | – | <0.0001 |
| Yes | 47 (15.99%) | 60 (20.41%) | 187 (63.61%) | – |
| No | 7,661 (27.82%) | 4,473 (16.24%) | 15,402 (55.93%) | – |
| **Primary Plan** |  |  |  | <0.0001 |
| Medicaid | 521 (24.19%) | 396 (18.38%) | 1,237 (57.43%) | – |
| Medicare | 3,131 (23.51%) | 2,668 (20.03%) | 7,519 (56.46%) | – |
| Private | 3,861 (33.16%) | 1,356 (11.65%) | 6,427 (55.20%) | – |
| Other | 79 (27.24%) | 51 (17.59%) | 160 (55.17%) | – |
| **Rural residence** ^c^ | – | – | – | <0.0001 |
| Yes | 1,474 (28.12%) | 1,021 (19.48%) | 2,747 (52.40%) | – |
| No | 6,191 (28.31%) | 3,380 (15.46%) | 12,294 (56.23%) | – |
| **Broadband access ^a,c^** | 81.98% | 80.19% | 81.52% | <0.0001 |
| **Below poverty threshold ^a,c^** | 13.14% | 14.25% | 13.80% | <0.0001 |
| **Income ^d^** | 65,841.06 | 61,286.43 | 64,059.48 | <0.0001 |
| **DDI ^d^** | 29.01 | 31.62 | 29.57 | <0.0001 |

**S4 Table.** Characteristics of patients seen in medical oncology (N=37,581) using each visit type

| **Variable** | **Video visit users**  **N=12,613** | **Phone visit users**  **N=11,062** | **Non-telehealth user**  **N=13,906** | **p-value** |
| --- | --- | --- | --- | --- |
| **Gender** | – | – | – | <0.0001 |
| Female | 7,306 (35.34%) | 5,766 (27.89%) | 7,603 (36.77%) | – |
| Male | 5,304 (31.38%) | 5,296 (31.34%) | 6,301 (37.28%) | – |
| **Age** ^a^ | 50.73 | 62.2 | 51.78 | <0.0001 |
| **Race** | – | – | – | <0.0001 |
| White or Caucasian | 10,704 (34.07%) | 9,358 (29.78%) | 11,360 (36.15%) | – |
| Black or African-American | 793 (28.82%) | 927 (33.68%) | 1,032 (37.50%) | – |
| Asian | 431 (40.28%) | 207 (19.35%) | 432 (40.37%) | – |
| All Others ^b^ | 338 (31.98%) | 286 (27.06%) | 433 (40.96%) | – |
| **Ethnicity** | – | – | – | 0.0007 |
| Non-Hispanic | 11,697 (33.86%) | 10,286 (29.78%) | 12,558 (36.36%) | – |
| Hispanic | 308 (34.38%) | 218 (24.33%) | 370 (41.29%) | – |
| **Needed Interpreter** | – | – | – | <0.0001 |
| Yes | 92 (22.33%) | 121 (29.37%) | 199 (48.30%) | – |
| No | 12,412 (33.63%) | 10,900 (29.53%) | 13,595 (36.84%) | – |
| **Primary Plan** | – | – | – | <0.0001 |
| Medicaid | 912 (30.02%) | 831 (27.35%) | 1,295 (42.63%) | – |
| Medicare | 4,064 (25.99%) | 6,211 (39.72%) | 5,361 (34.29%) | – |
| Private | 6,890 (41.10%) | 3,729 (22.24%) | 6,147 (36.66%) | – |
| Other | 69 (27.38%) | 78 (30.95%) | 105 (41.67%) | – |
| **Rural residence** ^c^ | – | – | – | 0.0014 |
| Yes | 1,844 (32.67%) | 1,768 (31.33%) | 2,032 (36.00%) | – |
| No | 10,503 (34.69%) | 8,830 (29.16%) | 10,944 (36.15%) | – |
| **Broadband access ^a,c^** | 82.60% | 81.56% | 81.90% | <0.0001 |
| **Below poverty threshold ^a,c^** | 12.96% | 13.53% | 13.43% | <0.0001 |
| **Income ^d^** | 67,147.45 | 64,464.81 | 65,272.93 | <0.0001 |
| **DDI ^d^** | 28.02 | 29.43 | 29.04 | <0.0001 |

**S5 Table.** Logistic regression models for all of oncology during Phase 1 of the COVID-19 pandemic (N=15,719 patients).

| **Variable** |  | **Estimate** | **Standard Error** | **p-value** |
| --- | --- | --- | --- | --- |
| Intercept |  | 1.4057 | 0.1088 | <0.0001 |
| Age |  | -0.037 | 0.00125 | <0.0001 |
| Gender | Female | 0.0173 | 0.0154 | 0.2607 |
| Race (ref=White) | Asian | 0.3174 | 0.0764 | <0.0001 |
|  | Black or African American | -0.2999 | 0.0599 | <0.0001 |
|  | All Others ^b^ | -0.1282 | 0.0893 | 0.1512 |
| Ethnicity | Hispanic | -0.087 | 0.0635 | 0.1703 |
| Rural |  | -0.2056 | 0.0535 | 0.0001 |
| DDI |  | -0.1018 | 0.0148 | <0.0001 |
| Primary Insurance (ref=Private) | Medicaid | -0.4164 | 0.0657 | <0.0001 |
|  | Medicare | 0.1562 | 0.056 | 0.0053 |
|  | Other | -0.0916 | 0.141 | 0.5161 |
| Interpreter |  | -0.1646 | 0.1721 | 0.3387 |
| Average Distance  (per 10 miles) |  | 0.0274 | 0.00358 | <0.0001 |

**S6 Table.** Odds ratios for all of oncology during Phase 1 of the COVID-19 pandemic (N=15,719 patients). Note that these data were used to generate Fig 3.

| **Variable** |  | **Odds Ratio** | **OR 95% Confidence Interval** | |
| --- | --- | --- | --- | --- |
| Age |  | 0.964 | 0.961 | 0.966 |
| Gender | Female | 1.035 | 0.975 | 1.099 |
| Race (ref=white) | Asian | 1.229 | 1.022 | 1.479 |
|  | Black or African American | 0.663 | 0.584 | 0.753 |
|  | All Others ^b^ | 0.787 | 0.629 | 0.986 |
| Ethnicity | Hispanic | 0.840 | 0.655 | 1.078 |
| Rural |  | 0.814 | 0.733 | 0.904 |
| DDI |  | 0.903 | 0.877 | 0.930 |
| Primary Insurance (ref=Private) | Medicaid | 0.464 | 0.410 | 0.525 |
|  | Medicare | 0.822 | 0.761 | 0.888 |
|  | Other | 0.642 | 0.444 | 0.929 |
| Interpreter |  | 0.848 | 0.605 | 1.188 |
| Average Distance  (per 10 miles) |  | 1.028 | 1.021 | 1.035 |

**S7 Table**. Logistic regression models for radiation oncology during Phase 1 of the COVID-19 pandemic (N=2,648 patients).

| **Variable** |  | **Estimate** | **Standard Error** | **p-value** |
| --- | --- | --- | --- | --- |
| Intercept |  | 0.2426 | 0.4132 | 0.5571 |
| Age |  | -0.0203 | 0.00506 | <0.0001 |
| Gender | Female | 0.0384 | 0.047 | 0.4141 |
| Race (ref=White) | Asian | 0.3053 | 0.2107 | 0.1473 |
|  | Black or African American | -0.3172 | 0.1888 | 0.0929 |
|  | All Others ^b^ | -0.108 | 0.2598 | 0.6775 |
| Ethnicity | Hispanic | 0.0447 | 0.2038 | 0.8264 |
| Rural |  | -0.2651 | 0.1672 | 0.1128 |
| DDI |  | -0.1341 | 0.0433 | 0.0020 |
| Primary Insurance (ref=Private) | Medicaid | -0.5969 | 0.2309 | 0.0097 |
|  | Medicare | 0.2508 | 0.1648 | 0.1281 |
|  | Other | -0.0618 | 0.3874 | 0.8733 |
| Interpreter |  | 0.7347 | 0.4347 | 0.0910 |
| Average Distance  (per 10 miles) |  | 0.0297 | 0.0109 | 0.0065 |

**S8 Table.** Odds ratios for radiation oncology during Phase 1 of the COVID-19 pandemic (N=2,648 patients). Note that these data were used to generate S1 Fig.

| **Variable** |  | **Odds Ratio** | **OR 95% Confidence Interval** | |
| --- | --- | --- | --- | --- |
| Age |  | 0.980 | 0.970 | 0.990 |
| Gender | Female | 1.080 | 0.898 | 1.298 |
| Race (ref=white) | Asian | 1.204 | 0.730 | 1.984 |
|  | Black or African American | 0.646 | 0.427 | 0.978 |
|  | All Others ^b^ | 0.796 | 0.414 | 1.531 |
| Ethnicity | Hispanic | 1.094 | 0.492 | 2.431 |
| Rural |  | 0.767 | 0.553 | 1.065 |
| DDI |  | 0.874 | 0.803 | 0.952 |
| Primary Insurance (ref=Private) | Medicaid | 0.366 | 0.217 | 0.617 |
|  | Medicare | 0.855 | 0.676 | 1.081 |
|  | Other | 0.625 | 0.227 | 1.719 |
| Interpreter |  | 2.085 | 0.889 | 4.888 |
| Average Distance  (per 10 miles) |  | 1.030 | 1.008 | 1.052 |

**S9 Table.** Logistic regression models for surgical oncology during Phase 1 of the COVID-19 pandemic (N=2,026 patients).

| **Variable** |  | **Estimate** | **Standard Error** | **p-value** |
| --- | --- | --- | --- | --- |
| Intercept |  | 0.9023 | 0.3799 | 0.0175 |
| Age |  | -0.0231 | 0.00484 | <0.0001 |
| Gender | Female | -0.0943 | 0.0434 | 0.0300 |
| Race (ref=White) | Asian | -0.0750 | 0.2453 | 0.7597 |
|  | Black or African American | -0.2331 | 0.1714 | 0.1738 |
|  | All Others ^b^ | 0.0229 | 0.2662 | 0.9314 |
| Ethnicity | Hispanic | -0.3844 | 0.2218 | 0.0831 |
| Rural |  | -0.2794 | 0.1432 | 0.051 |
| DDI |  | -0.1444 | 0.0421 | 0.0006 |
| Primary Insurance (ref=Private) | Medicaid | -0.5242 | 0.1840 | 0.0044 |
|  | Medicare | 0.00155 | 0.1601 | 0.9923 |
|  | Other | 0.1331 | 0.3985 | 0.7384 |
| Interpreter |  | 0.0761 | 0.4409 | 0.8629 |
| Average Distance  (per 10 miles) |  | 0.0406 | 0.00962 | <0.0001 |

**S10 Table.** Odds ratios for surgical oncology during Phase 1 of the COVID-19 pandemic (N=2,026 patients). Note that these data were used to generate S2 Fig.

| **Variable** |  | **Odds Ratio** | **OR 95% Confidence Interval** | |
| --- | --- | --- | --- | --- |
| Age |  | 0.977 | 0.968 | 0.987 |
| Gender | Female | 0.828 | 0.698 | 0.982 |
| Race (ref=white) | Asian | 0.697 | 0.381 | 1.277 |
|  | Black or African American | 0.596 | 0.425 | 0.835 |
|  | All Others ^b^ | 0.769 | 0.394 | 1.501 |
| Ethnicity | Hispanic | 0.464 | 0.194 | 1.106 |
| Rural |  | 0.756 | 0.571 | 1.001 |
| DDI |  | 0.866 | 0.797 | 0.940 |
| Primary Insurance (ref=Private) | Medicaid | 0.401 | 0.284 | 0.566 |
|  | Medicare | 0.678 | 0.538 | 0.855 |
|  | Other | 0.774 | 0.273 | 2.192 |
| Interpreter |  | 1.079 | 0.455 | 2.560 |
| Average Distance  (per 10 miles) |  | 1.041 | 1.022 | 1.061 |

**S11 Table.** Logistic regression models for medical oncology during Phase 1 of the COVID-19 pandemic (N=12,222 patients).

| **Variable** |  | **Estimate** | **Standard Error** | **p-value** |
| --- | --- | --- | --- | --- |
| Intercept |  | 1.5825 | 0.1215 | <0.0001 |
| Age |  | -0.0405 | 0.00137 | <0.0001 |
| Gender | Female | 0.0244 | 0.0177 | 0.169 |
| Race (ref=White) | Asian | 0.3684 | 0.087 | <0.0001 |
|  | Black or African American | -0.3618 | 0.0681 | <0.0001 |
|  | All Others ^b^ | -0.0875 | 0.1008 | 0.3849 |
| Ethnicity | Hispanic | -0.0748 | 0.0695 | 0.2816 |
| Rural |  | -0.2156 | 0.0616 | 0.0005 |
| DDI |  | -0.0973 | 0.0171 | <0.0001 |
| Primary Insurance (ref=Private) | Medicaid | -0.4201 | 0.0758 | <0.0001 |
|  | Medicare | 0.1743 | 0.0659 | 0.0081 |
|  | Other | -0.1276 | 0.1673 | 0.4455 |
| Interpreter |  | -0.441 | 0.2086 | 0.0345 |
| Average Distance  (per 10 miles) |  | 0.0243 | 0.00417 | <0.0001 |

**S12 Table.** Odds ratios for medical oncology during Phase 1 of the COVID-19 pandemic (N=12,222 patients). Note that these data were used to generate S3 Fig.

| **Variable** |  | **Odds Ratio** | **OR 95% Confidence Interval** | |
| --- | --- | --- | --- | --- |
| Age |  | 0.960 | 0.958 | 0.963 |
| Gender | Female | 1.050 | 0.979 | 1.126 |
| Race (ref=white) | Asian | 1.333 | 1.080 | 1.645 |
|  | Black or African American | 0.642 | 0.556 | 0.742 |
|  | All Others ^b^ | 0.845 | 0.656 | 1.089 |
| Ethnicity | Hispanic | 0.861 | 0.656 | 1.131 |
| Rural |  | 0.806 | 0.714 | 0.910 |
| DDI |  | 0.907 | 0.877 | 0.938 |
| Primary Insurance (ref=Private) | Medicaid | 0.452 | 0.394 | 0.519 |
|  | Medicare | 0.819 | 0.750 | 0.895 |
|  | Other | 0.606 | 0.391 | 0.939 |
| Interpreter |  | 0.643 | 0.427 | 0.968 |
| Average Distance  (per 10 miles) |  | 1.025 | 1.016 | 1.033 |

**S13 Table.** Logistic regression models for all of oncology during Phase 2 of the COVID-19 pandemic (N=6,716 patients).

| **Variable** |  | **Estimate** | **Standard Error** | **p-value** |
| --- | --- | --- | --- | --- |
| Intercept |  | 2.7704 | 0.1864 | <0.0001 |
| Age |  | -0.0299 | 0.00226 | <0.0001 |
| Gender | Female | -0.00994 | 0.0262 | 0.7047 |
| Race (ref=White) | Asian | 0.402 | 0.151 | 0.0078 |
|  | Black or African American | -0.4485 | 0.0974 | <0.0001 |
|  | All Others ^b^ | -0.0166 | 0.1448 | 0.9088 |
| Ethnicity | Hispanic | -0.1317 | 0.0962 | 0.1709 |
| Rural |  | -0.2756 | 0.0859 | 0.0013 |
| DDI |  | -0.1303 | 0.0246 | <0.0001 |
| Primary Insurance (ref=Private) | Medicaid | -0.4322 | 0.1071 | <0.0001 |
|  | Medicare | 0.1236 | 0.0911 | 0.1749 |
|  | Other | -0.3011 | 0.2271 | 0.1849 |
| Interpreter |  | 0.0409 | 0.3224 | 0.8991 |
| Average Distance  (per 10 miles) |  | 0.0365 | 0.00612 | <0.0001 |

**S14 Table.** Odds ratios for all of oncology during Phase 2 of the COVID-19 pandemic (N=6,716 patients). Note that these data were used to generate Fig 3.

| **Variable** |  | **Odds Ratio** | **OR 95% Confidence Interval** | |
| --- | --- | --- | --- | --- |
| Age |  | 0.971 | 0.966 | 0.975 |
| Gender | Female | 0.980 | 0.885 | 1.086 |
| Race (ref=white) | Asian | 1.403 | 0.962 | 2.047 |
|  | Black or African American | 0.600 | 0.494 | 0.728 |
|  | All Others ^b^ | 0.923 | 0.644 | 1.323 |
| Ethnicity | Hispanic | 0.768 | 0.527 | 1.120 |
| Rural |  | 0.759 | 0.642 | 0.898 |
| DDI |  | 0.878 | 0.837 | 0.921 |
| Primary Insurance (ref=Private) | Medicaid | 0.353 | 0.287 | 0.434 |
|  | Medicare | 0.615 | 0.535 | 0.707 |
|  | Other | 0.402 | 0.222 | 0.730 |
| Interpreter |  | 1.042 | 0.554 | 1.960 |
| Average Distance  (per 10 miles) |  | 1.037 | 1.025 | 1.050 |

**S15 Table.** Logistic regression models for radiation oncology during Phase 2 of the COVID-19 pandemic (N=1,124 patients).

| **Variable** |  | **Estimate** | **Standard Error** | **p-value** |
| --- | --- | --- | --- | --- |
| Intercept |  | 3.9141 | 0.7938 | <0.0001 |
| Age |  | -0.0438 | 0.00931 | <0.0001 |
| Gender | Female | -0.2391 | 0.0752 | 0.0015 |
| Race (ref=White) | Asian | -0.2183 | 0.3473 | 0.5297 |
|  | Black or African American | -0.1408 | 0.2671 | 0.598 |
|  | All Others ^b^ | 0.0876 | 0.4632 | 0.85 |
| Ethnicity | Hispanic | -0.2072 | 0.3377 | 0.5396 |
| Rural |  | -0.0022 | 0.2486 | 0.9929 |
| DDI |  | -0.1427 | 0.0678 | 0.0352 |
| Primary Insurance (ref=Private) | Medicaid | -0.884 | 0.4726 | 0.0614 |
|  | Medicare | -0.1115 | 0.4234 | 0.7922 |
|  | Other | 0.7767 | 1.1886 | 0.5134 |
| Interpreter |  | 1.7036 | 0.9692 | 0.0788 |
| Average Distance  (per 10 miles) |  | 0.0475 | 0.019 | 0.0125 |

**S16 Table.** Odds ratios for radiation oncology during Phase 2 of the COVID-19 pandemic (N=1,124 patients). Note that these data were used to generate S1 Fig.

| **Variable** |  | **Odds Ratio** | **OR 95% Confidence Interval** | |
| --- | --- | --- | --- | --- |
| Age |  | 0.957 | 0.940 | 0.975 |
| Gender | Female | 0.620 | 0.462 | 0.832 |
| Race (ref=white) | Asian | 0.613 | 0.269 | 1.393 |
|  | Black or African American | 0.662 | 0.392 | 1.119 |
|  | All Others ^b^ | 0.832 | 0.255 | 2.719 |
| Ethnicity | Hispanic | 0.661 | 0.176 | 2.483 |
| Rural |  | 0.998 | 0.613 | 1.624 |
| DDI |  | 0.867 | 0.759 | 0.990 |
| Primary Insurance (ref=Private) | Medicaid | 0.332 | 0.166 | 0.666 |
|  | Medicare | 0.719 | 0.475 | 1.088 |
|  | Other | 1.747 | 0.078 | 39.206 |
| Interpreter |  | 5.493 | 0.822 | 36.715 |
| Average Distance  (per 10 miles) |  | 1.049 | 1.010 | 1.088 |

**S17 Table.** Logistic regression models for surgical oncology during Phase 2 of the COVID-19 pandemic (N=752 patients).

| **Variable** |  | **Estimate** | **Standard Error** | **p-value** |
| --- | --- | --- | --- | --- |
| Intercept |  | 3.7555 | 0.8459 | <0.0001 |
| Age |  | -0.0295 | 0.00922 | 0.0014 |
| Gender | Female | 0.1547 | 0.084 | 0.0656 |
| Race (ref=White) | Asian | 0.4046 | 0.7269 | 0.5778 |
|  | Black or African American | 0.0579 | 0.3735 | 0.8768 |
|  | All Others ^b^ | -0.5143 | 0.4571 | 0.2606 |
| Ethnicity | Hispanic | 0.5148 | 0.5524 | 0.3514 |
| Rural |  | -0.5121 | 0.2414 | 0.0339 |
| DDI |  | -0.2728 | 0.0782 | 0.0005 |
| Primary Insurance (ref=Private) | Medicaid | -0.1786 | 0.3171 | 0.5732 |
|  | Medicare | 0.3197 | 0.2434 | 0.1890 |
|  | Other | -0.8884 | 0.5349 | 0.0968 |
| Interpreter |  | -1.3361 | 1.1337 | 0.2386 |
| Average Distance  (per 10 miles) |  | 0.0512 | 0.0179 | 0.0042 |

**S18 Table.** Odds ratios for surgical oncology during Phase 2 of the COVID-19 pandemic (N=752 patients). Note that these data were used to generate S2 Fig.

| **Variable** |  | **Odds Ratio** | **OR 95% Confidence Interval** | |
| --- | --- | --- | --- | --- |
| Age |  | 0.971 | 0.954 | 0.989 |
| Gender | Female | 1.363 | 0.980 | 1.894 |
| Race (ref=white) | Asian | 1.423 | 0.221 | 9.158 |
|  | Black or African American | 1.006 | 0.503 | 2.013 |
|  | All Others ^b^ | 0.568 | 0.206 | 1.563 |
| Ethnicity | Hispanic | 2.800 | 0.321 | 24.410 |
| Rural |  | 0.599 | 0.373 | 0.962 |
| DDI |  | 0.761 | 0.653 | 0.887 |
| Primary Insurance (ref=Private) | Medicaid | 0.396 | 0.193 | 0.814 |
|  | Medicare | 0.652 | 0.417 | 1.020 |
|  | Other | 0.195 | 0.048 | 0.789 |
| Interpreter |  | 0.263 | 0.028 | 2.425 |
| Average Distance  (per 10 miles) |  | 1.053 | 1.016 | 1.090 |

**S19 Table.** Logistic regression models for medical oncology during Phase 2 of the COVID-19 pandemic (N=5,365 patients).

| **Variable** |  | **Estimate** | **Standard Error** | **p-value** |
| --- | --- | --- | --- | --- |
| Intercept |  | 2.7388 | 0.2031 | <0.0001 |
| Age |  | -0.0307 | 0.00241 | <0.0001 |
| Gender | Female | 0.0265 | 0.0296 | 0.3698 |
| Race (ref=White) | Asian | 0.4954 | 0.1711 | 0.0038 |
|  | Black or African American | -0.5448 | 0.1086 | <0.0001 |
|  | All Others ^b^ | -0.0107 | 0.1586 | 0.9463 |
| Ethnicity | Hispanic | -0.114 | 0.1023 | 0.2653 |
| Rural |  | -0.3051 | 0.0979 | 0.0018 |
| DDI |  | -0.1037 | 0.0277 | 0.0002 |
| Primary Insurance (ref=Private) | Medicaid | -0.4613 | 0.1216 | 0.0001 |
|  | Medicare | 0.0905 | 0.1068 | 0.3972 |
|  | Other | -0.2325 | 0.271 | 0.391 |
| Interpreter |  | -0.1289 | 0.3676 | 0.7258 |
| Average Distance  (per 10 miles) |  | 0.0334 | 0.00685 | <0.0001 |

**S20 Table.** Odds ratios for medical oncology during Phase 2 of the COVID-19 pandemic (N=5,365 patients). Note that these data were used to generate S3 Fig.

| **Variable** |  | **Odds Ratio** | **OR 95% Confidence Interval** | |
| --- | --- | --- | --- | --- |
| Age |  | 0.970 | 0.965 | 0.974 |
| Gender | Female | 1.055 | 0.939 | 1.184 |
| Race (ref=white) | Asian | 1.546 | 1.007 | 2.373 |
|  | Black or African American | 0.546 | 0.439 | 0.679 |
|  | All Others ^b^ | 0.932 | 0.630 | 1.378 |
| Ethnicity | Hispanic | 0.796 | 0.533 | 1.189 |
| Rural |  | 0.737 | 0.608 | 0.893 |
| DDI |  | 0.901 | 0.854 | 0.952 |
| Primary Insurance (ref=Private) | Medicaid | 0.345 | 0.276 | 0.431 |
|  | Medicare | 0.599 | 0.512 | 0.700 |
|  | Other | 0.434 | 0.213 | 0.883 |
| Interpreter |  | 0.879 | 0.428 | 1.807 |
| Average Distance  (per 10 miles) |  | 1.034 | 1.020 | 1.048 |
